# Editorial: Understanding the effects of transcranial current stimulation on the locomotor and musculoskeletal systems

**DOI:** 10.3389/fnhum.2023.1189405

**Published:** 2023-04-11

**Authors:** Stephane Perrey, Shawn D. Flanagan

**Affiliations:** ^1^EuroMov Digital Health in Motion, University of Montpellier, IMT Mines Ales, Montpellier, France; ^2^Neuromuscular Research Laboratory, University of Pittsburgh, Pittsburgh, PA, United States

**Keywords:** transcranial current direct stimulation, locomotor, sensorimotor function, cerebellum, primary motor cortex

## 1. Introduction

Over the last decade, there has been growing interest in the use of non-invasive brain stimulation techniques such as transcranial current stimulation (tCS) to improve physical performance and locomotor functions. In essence, tCS delivers weak currents to the cortex transcutaneously, with active electrode(s) placed on the area(s) of the scalp overlying the cortical target(s) and reference electrode(s) placed elsewhere, traditionally over the contralateral supraorbital area or a non-cephalic location. Electrical currents that reach the cortex can transiently depolarize or hyperpolarize neuronal resting membrane potentials and thereby alter excitability and action potential generation probability. The majority of tCS studies target the primary motor cortex (M1), which plays important roles in the acquisition and execution of locomotor and musculoskeletal functions. It is thought that M1 tCS can facilitate various aspects of motor function such as movement precision, learning, strength, and endurance. Notably, some of these improvements include hand and leg functions used in activities of daily living (Morya et al., 2019). Accordingly, in addition to investigation as a therapeutic modality to improve locomotor and musculoskeletal functions affected by various neurological disorders, M1 tCS is increasingly used to enhance motor functions in healthy individuals (Maudrich et al., 2022). Given the distributed nature of the sensorimotor network and widely ranging nature of neurological motor impairments however, it is necessary to assess the feasibility and efficacy of tCS targeted to other sensorimotor neuronal structures. For example, the cerebellum plays a pivotal role in the development and synchronization of reaching and movement tasks, and is thus an attractive target for therapeutic tCS. This *Frontiers in Human Neuroscience: Brain Imaging and Stimulation* Research Topic includes six contributions (four original and two case reports) from a total of 28 co-authors. Efforts were centered around the effects of tCS when applied to M1 or cerebellum to improve upper- or lower-extremity locomotor or musculoskeletal functions in healthy individuals or clinical populations.

## 2. Effects of M1 tCS on motor function

Motor cortex tCS can facilitate contralateral unimanual movements (Krishnan et al., 2014). However, because most physical activities involve coordinated whole-body movements, tCS-induced functional improvements in one part of the body (e.g., right first dorsal interosseous abduction force) may not translate to other areas of the body or result in paradoxical compensatory adaptations (e.g., functional impairments in other muscles). Hikosaka and Aramaki examined the effects of transcranial direct current stimulation (tDCS) on bimanual handgrip strength, which is difficult to predict because of complex neural dynamics that involve interhemispheric interactions and ipsilateral projections. Their results revealed that bimanual handgrip strength improved with a left M1 anode - right M1 cathode electrode placement montage, and not with a right M1 anode – left M1 cathode montage or sham stimulation. These findings provide important preliminary evidence to suggest that with specific e-field patterns, M1 tDCS can improve bilateral upper limb functions in healthy individuals.

Most tDCS studies use currents of 1-2 mA, but higher intensities are safe and tolerable (Nitsche and Bikson, 2017). Critically, if ~ 1 mV · mm^−1^ is needed to induce neuromodulatory effects (Voroslakos et al., 2018) given ~ 80% current loss at the scalp, 4-6 mA may be required to reliably alter neuronal circuits and co-dependent functional outcomes. Compared with sham and 4 mA stimulation and contrary to previous *submaximal* isometric fatigue investigations, Workman et al. showed that 2 mA tDCS of the M1 contralateral to the dominant leg reduced knee extensor torque and EMG activity during a *maximal* isokinetic fatigue task. When compared with sham, 4 mA did not induce differential effects on knee torque or EMG activity. Workman et al. results suggest that the behavioral aftereffects of tDCS may not scale linearly with stimulation intensity. In addition to the timing of stimulation, these results also support growing evidence that the effects of tDCS may depend on past and current activity in stimulated neuronal structures. Ultimately, the effects of higher intensity (> 2 mA) tDCS on sensorimotor functions in healthy individuals and clinical populations must be further explored.

In a case report by Shima, Miyake et al. a combination of gait-synchronized (swing phase) closed-loop brain stimulation and robot-assisted knee joint training was used to improve post-stroke gait disturbance in a 57-year-old patient with chronic post-stroke hemiparesis. Transcranial alternating current stimulation (tACS) was applied to the lesioned M1 foot area with a 1.5 mA positive DC offset and 3 mA sine-wave current triggered by paretic heel contact to deliver maximum current immediately before the swing phase. The combination of closed-loop stimulation and robot training improved 10-meter gait speed, timed up and go time, and lower limb muscle strength. Improvements in gait and balance functions persisted 2 weeks after the intervention.

## 3. Effects of cerebellar tCS on sensorimotor function

In situations where complex environments introduce delays between actions and sensory outcomes, flexible recalibration of the corresponding temporal predictions is required. While the cerebellum has long been associated with sensorimotor functions, its relative contributions to sensorimotor and inter-sensory temporal recalibration are unknown. Schmitter and Straube used cerebellar tDCS as a causal probe to assess the role of the cerebellum in these processes. Compared with cathodal, dual-hemisphere, and sham cerebellar tDCS, changes in sensorimotor recalibration after anodal cerebellar tDCS indicate a generalized role of the cerebellum in temporal recalibration across sensorimotor and perceptual domains. In addition, differences in temporal recalibration effects across delay levels suggest that cerebellar-dependent sensorimotor temporal recalibration processes are probable and may depend on the availability of action-based predictions.

Multiple sclerosis (MS) is a central nervous system inflammatory disease that involves demyelination and neurodegeneration. The complexity of the disease and heterogeneous nature of clinical symptoms and progression necessitates tailored approaches that combine pharmaceutical and physical rehabilitation therapies. The non-invasive modulation of cerebellar activity may be useful as an adjunct therapy to improve gait function in individuals with MS. Using a split-belt treadmill paradigm, Nguemeni et al. examined the effect of ipsilateral (to fast leg) anodal 2 mA cerebellar tDCS on locomotor adaptation and consolidation in 40 individuals with mild-to-moderate MS and 30 healthy controls. Locomotor adaptation and consolidation did not differ between groups, and the immediate post-task application of anodal cerebellar tDCS did not improve adaptation or consolidation compared with sham. In addition to suggesting that locomotor adaptation capacity is maintained in mild-to-moderate MS, these findings highlight the importance of efforts to further optimize cerebellar tDCS parameters such as timing and intensity.

In another case report, Shima, Tanaka et al. presented the results of a backward gait training intervention combined with synchronized cerebellar tACS in an individual with progressive supranuclear palsy-Richardson's syndrome, which is characterized by postural instability. tACS was applied to the right cerebellum in accordance with the lateralized severity of motor symptoms. Stimulation was delivered at 3 mA and timed to coincide with right leg backward gait frequency. In contrast to asynchronized or sham stimulation, synchronized stimulation reduced postural instability, and long-term stimulation further improved postural stability and related outcomes such as gait speed, balance function, and fall-related self-efficacy. These preliminary results support continued efforts to develop non-invasive brain stimulation protocols that leverage endogenous physiological activity to enhance therapeutic efficacy.

## 4. Conclusion and perspectives

What near-term advances can we expect in the use of tCS to improve locomotor and musculoskeletal system function in health and disease? As highlighted by the work included in this Research Topic, efforts to identify the most effective and safe stimulation parameters will continue. Such efforts will be aided by machine learning techniques that enable the *real-time* analysis of complex sensorimotor signals with unparalleled sensitivity. Machine learning techniques will also benefit efforts to improve tCS efficacy by “closing the loop” with better stimulus timing accuracy and the identification of more effective physiological “triggers”. In addition to acute effects, concerted efforts to understand the effects of repeated tCS are needed. To improve tCS efficacy, especially when combined with locomotor or musculoskeletal behaviors and therapies, the nature of tCS interactions with past and current physiological activity must be further elucidated. Similarly, while progress has been made, there is a need to further emphasize the investigation of tCS in the target clinical populations.

## Author contributions

SP and SF wrote and revised the final version of the manuscript. Both authors have read and approved the final submitted version.
